# Visualization of the research landscape on the 20 m shuttle run test in the last 15 years: bibliometric analysis with VOSviewer of the most cited studies

**DOI:** 10.3389/fspor.2025.1568501

**Published:** 2025-05-15

**Authors:** Boryi A. Becerra-Patiño, Juan David Paucar Uribe, Rodrigo Yáñez-Sepúlveda, Tomas Reyes-Amigo, Júlio Brugnara Mello, João Martins, Anelise Reis Gaya, José Francisco López-Gil, Jorge Olivares-Arancibia

**Affiliations:** ^1^ Faculty of Physical Education, Bachelor’s Degree in Sports, National Pedagogical University, Bogotá, Colombia; ^2^ Management and Pedagogy of Physical Activity and Sport (GPAFD), Faculty of Physical Education, National Pedagogical University, Bogotá, Colombia; ^3^ Faculty Education and Social Sciences, Universidad Andres Bello, Viña del Mar, Chile; ^4^ Physical Activity Sciences Observatory (OCAF), Department of Physical Activity Sciences, Universidad de Playa Ancha, Valparaiso, Chile; ^5^ eFiDac Research Group, School of Physical Education, Pontificia Universidad Católica de Valparaíso, Valparaíso, Chile; ^6^ Centro de Estudos em Educacão, Faculdade de Motricidade Humana e UIDEF, Universidade de Lisboa, Lisboa, Portugal; ^7^ Projeto Esporte Brasil (PROESP-Br), School of Physical Education, Physiotherapy and Dance, Post-graduation Program in Human Movement Sciences, Federal University of Rio Grande do Sul, Porto Alegre, Brazil; ^8^ School of Medicine, Universidad Espíritu Santo, Samborondón, Ecuador; ^9^ Vicerrectoría de Investigación y Postgrado, Universidad de Los Lagos, Osorno, Chile; ^10^ AFySE Group, Research in Physical Activity and School Health, School of Physical Education, Faculty of Education, Universidad de las Américas, Santiago, Chile

**Keywords:** maximum oxygen consumption, multistage physical fitness test, aerobic fitness, cardiorespiratory endurance, youth, childhood

## Abstract

**Introduction:**

Various studies have applied the 20-meter shuttle run test (20mSRT) to estimate cardiorespiratory fitness in different population groups, with the aim of associating test performance with the physiological, psychosocial, and cognitive health of children and young people. However, to date, no bibliometric review that analyzes the research landscape has been conducted. The objective was to conduct a bibliometric review to develop an overview of the current state of scientific literature and identify research trends in the study of the 20mSRT in the Web of Science and PubMed databases.

**Methods:**

The final sample consisted of 797 documents. For the publication period, between 2010 and 2021, there was a considerable increase of 248.1% of the number of investigations. The year 2011 had the highest citation count, and the trend reveals low citation rates for the years 2018 and 2023, with decreases of −70.40% and −86.65% compared with the years 2012 and 2019, respectively.

**Results:**

Most of the production is in research articles (95.98%). The most cited authors are Ruiz, Castro-Piñero, Mayorga-Vega, Ortega, and Tomkinson. The five concepts with the highest occurrence in the research are children (*n* = 290), cardiorespiratory fitness (*n* = 205), adolescents (*n* = 203), performance (*n* = 164), and aerobic fitness (*n* = 146). The journals with the most published documents are the Journal of Strength and Conditioning Research (*n* = 45), the International Journal of Environmental Research and Public Health (*n* = 42), and the Journal of Sports Sciences (*n* = 25). The countries with the highest number of published documents and citations are Spain (*n* = 187 documents and 6,209 citations), the United States (*n* = 112 documents and 3,475 citations), Australia (*n* = 85 documents and 2,393 citations), and Sweden (*n* = 54 documents and 4,121 citations).

**Discussion:**

An analysis of the existing knowledge produced by the 20mSRT revealed that there is a preference for studying the school-age stage, with cardiorespiratory fitness variables associated with physical activity and sedentary time. Finally, there are different applications of the 20mSRT in various population groups, ranging from the evaluation of cardiorespiratory fitness in children, adolescents, and adults, as well as in specific groups of athletes.

## 1 Introduction

Physical fitness is related to morbidity and mortality (1). Moreover, engaging in moderate to vigorous intensity physical activity improves cardiorespiratory fitness, provides additional benefits in terms of all-cause mortality (2). Among the components of physical fitness, cardiorespiratory fitness (CRF) has been shown to represent the greatest benefit and is inversely associated with long-term mortality (3). Various studies highlight the importance of physical activity for health (4) and the assessment of physical fitness via tests that are easy to administer and highly valid.

An association has been established between low physical fitness and higher cardiometabolic risk in children, making it relevant to consider assessments with internationally validated tests (5). Various tests are used to evaluate CRF, but the 20-m shuttle run test (20mSRT) is one of the most widely used tests and has been validated and applied in different contexts and populations. This makes a bibliometric review a good strategy to analyze studies that have utilized this test. This test allows for the evaluation of CRF in both sports and educational settings (6). Nevertheless, the classification and application of these methods in different groups have been the subject of study and debate.

In this sense, another contribution of the present bibliometric analysis is that it details the areas of knowledge and variables, in this case sports, in which the 20mSRT has been most used, which allows us to identify the topics that need more exploration. These results are associated with those reported in the study by van der Zwaard et al. (7) where it has been reported that articles on exercise, training, performance and V˙o2max and large collaborative articles in contrast to articles on respiratory physiology or sleep apnea and letters to the editor. These findings serve as a reference for rethinking new lines of research. On the other hand, various studies have demonstrated good reliability of the test for quantifying VO_2max_ in adolescents and adults (8). In addition to its reliability, this test can be applied in various settings, such as educational contexts (9). Its easy application and standardization across diverse populations make it a valuable evaluative tool. Therefore, the objective was to conduct a bibliometric review to develop a general description of the current state of the scientific literature and identify research trends in the study of the 20mSRT in the Web of Science and PubMed databases, including documents published from January 1, 2010, to July 8, 2024.

## 2 Material and methods

### 2.1 Design

This study uses bibliometrics as a research technique. According to Hernández-Torrano & Ho (10), bibliometric analysis is considered a theoretical study because it aims to systematize, analyze, and condense a large amount of data related to the temporality of studies, as well as their evolution over the years. This type of study seeks to examine information related to knowledge production in a specific area through specific criteria such as (i) authors; (ii) countries; (iii) institutions; (iv) keywords; (v) document citations; (vi) cocitations among cited references; and (vii) editorial groups (11).

### 2.2 Data extraction

The search for documents was conducted between July 6 and 8, 2024, by the two principal investigators in the Web of Science (WoS) database. WoS was used because it is the most commonly used database for developing bibliometric studies (12–14). A search was also conducted in the PubMed database. During the screening of the information, 93% of the documents were duplicates. Consequently, it was decided to consider only WoS studies and to add the papers that were not included. The WoS database has been considered in other bibliometric studies on Sport Sciences and following the guidelines of other bibliometric studies (15–17).

**Figure 1:**
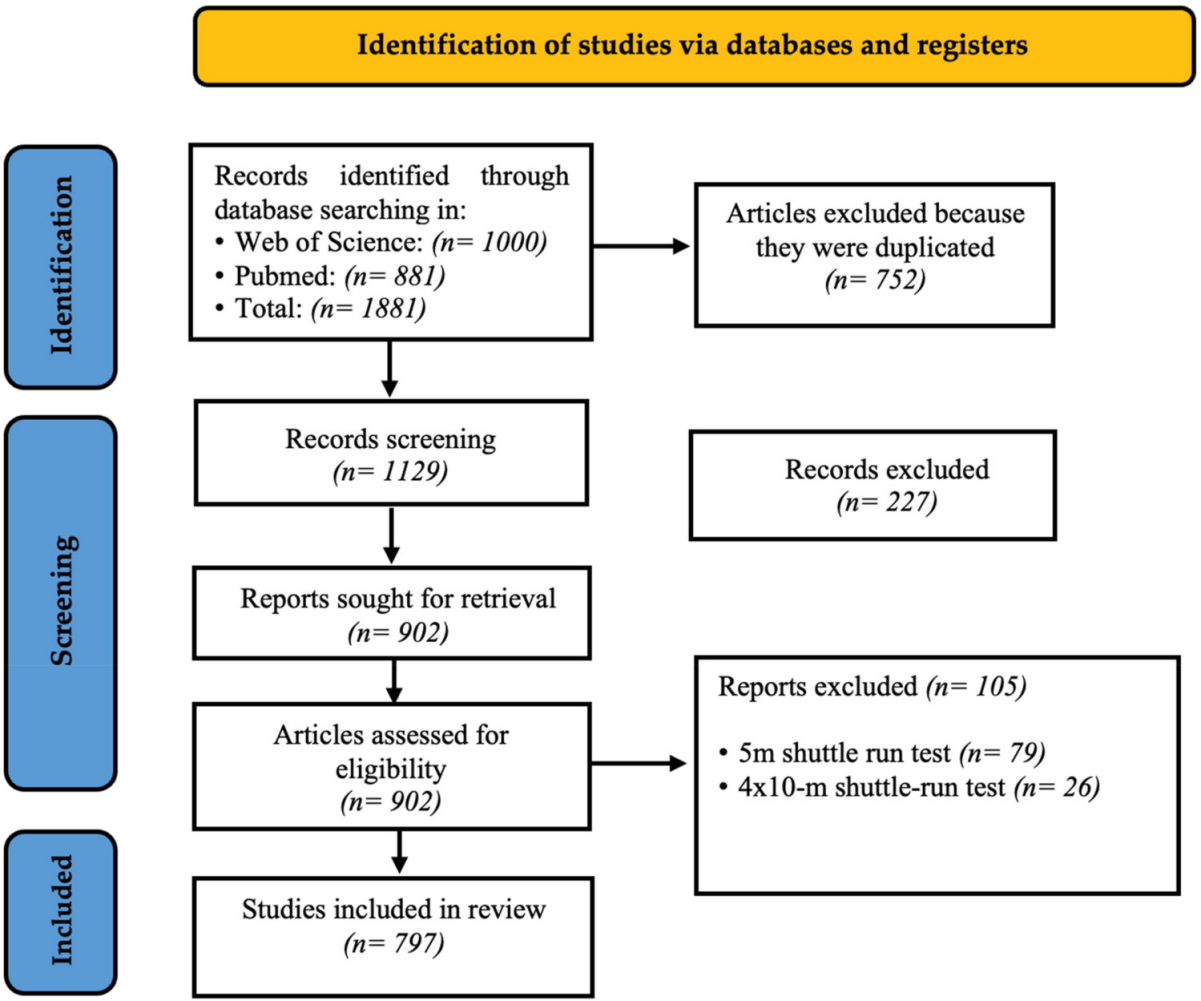
Flowchart for the selection of studies according to PRISMA guidelines.

A review was conducted on the basis of Preferred Reporting Items for Systematic reviews and Meta-Analyses (PRISMA) recommendations in the WoS database via the following terms: [“20 m shuttle run” (Title/Abstract)] OR [“20-m shuttle run test” (Title/Abstract)] OR [“20-meter shuttle run test” (Title/Abstract)] OR [“20mSRT” (Title/Abstract)]. After each of the concepts was reviewed, it was determined which one yielded the most documents. Finally, the search equation used was 20 m AND shuttle AND run. Three search periods were established between July 6 and 8, 2024 (Table 1). Each search used different terms, as there are various denominations for the “20mSRT” (Table 1). The search equation with the highest number of documents found was determined. These results were analyzed by two of the principal investigators (J.D.P.-U. and B.A.B.-P.). A systematic process was developed for selecting the documents included on the basis of bibliometric criteria such as document duplication and direct relevance to the study objective (18). Initially, 1,000 documents were analyzed and subjected to the metadata regulation process to eliminate duplicates, lack of full access, or lack of relevance to the study topic. After the PRISMA protocol was applied, 797 documents met the eligibility criteria established for this study (Figure 1).

**Table 1:** Overview of searches with keywords, Boolean terms, and the number of documents by type in the Web of science and pubMed databases.

N° search	Boolean operators	Search date	Publish date range	Total number of items found	Type of documents	Number of documents by type
1	“20-meter AND shuttle AND run AND test”	July 6, 2024	2010–2024	146	Article	138
					Review Article	2
					Letter	3
					Meeting Abstract	2
					Editorial	1
2	“20-m AND shuttle AND run AND test”	July 6, 2024	2010–2024	715	Article	684
					Review Article	25
					Letter	3
					Meeting Abstract	2
					Editorial	1
3	“20 m AND shuttle AND run”	July 7, 2024	2010–2024	1,000^a^	Article	962
					Review Article	29
					Letter	4
					Meeting Abstract	4
					Editorial	1
4	“20 m AND SRT”	July 7, 2024	2010–2024	10	Article	9
					Review Article	1
					Letter	0
					Meeting Abstract	0
					Editorial	0
5	“20-m AND endurance AND shuttle AND run AND test”	July 8, 2024	2010–2024	140	Article	130
					Review Article	10
					Letter	0
					Meeting Abstract	0
					Editorial	0
6	“20-m AND multistage AND shuttle AND run AND test”	July 8, 2024	2010–2024	94	Article	91
					Review Article	3
					Letter	0
					Meeting Abstract	0
					Editorial	0

^a^ This search was selected to perform the flowchart because it was the one with the highest number of records found.

### 2.3 Eligibility criteria

The inclusion criteria for the studies were as follows: (i) studies focused on the study of children, adolescents, or older adults; (ii) studies on samples of high-performance athletes, recreational athletes, or physically inactive individuals; (iii) original full-text studies reviewed by academic peers; (iv) access to the full document to complement the initial review; (v) documents written in English, Spanish, Portuguese, German, French, or Russian; (vi) publication date between January 1, 2010, and July 8, 2024; and (vii) journals indexed in the Journal Citation Report (JCR) in the quartiles: Q1, Q2, Q3, and Q4.

To select the documents, an analysis matrix was developed in Microsoft Excel based on the following categories: (1) year of publication; (2) type of document; (3) most cited articles; (4) authors’ names; (5) number of authors per study; (6) keywords; (7) journals and cited references; (8) editorial groups; (9) areas of knowledge; (10) language; (11) countries; (12) organizations; and (13) evaluated sample.

### 2.4 Data analysis

The data found in the WoS database were extracted in two different formats: plain text and Excel. This action allows for descriptive and percentage analyses of the results via a Microsoft Excel spreadsheet (v. 2006, Microsoft Corporation, Redmond, WA, USA), and, similarly, the download of data in plain text format enables the development of analyses by coauthorship of authors, coauthorship by organizations, occurrence by keywords, citation by documents, citation by journals, citation by countries, and citation by authors via the VOSviewer program (v.6.19., Center for Science and Technology Studies, Netherlands). The attraction and repulsion parameters in VOSviewer allow for the creation of maps in response to the topic. The parameters of 3 and −3 were addressed, following those of other recently published bibliometric analyses (19, 20) on the basis of nodes, seeking to identify the relationships established in scientific production from the information stored in various databases (21). Additionally, to strengthen the bibliometric analysis, the following laws were considered: (i) Price's law using the *R*^2^ coefficient (22); (ii) Lotka law to identify the authors with the most publications (23, 24); and (iii) Zipf law to identify the occurrence of the most used terms (25). The *h*-index was also used to determine the academic productivity of the authors, aiming to establish that *h* documents have been cited at least a minimum *h* of times (26).

## 3 Results

### 3.1 Evolution of the number of documents

The analysis of scientific production reveals an increasing number of publications per year. There is oscillatory growth from the first year analyzed to the last year, with 2020, 2021, and 2022 being the years with the highest production (*n* = 259). Likewise, it is revealed that by mid-2024, the trend reflects a low number of published documents.

**Figure 2:**
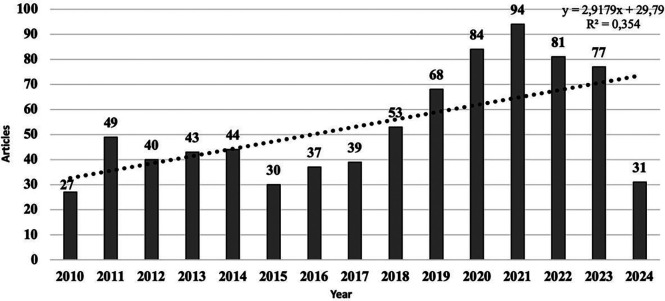
Evolution of the number of annual publications.

With respect to the *R*^2^ coefficient of the sample, exponential growth of the sample was identified, considering the number of publications from 2010 to 2018, at 96.29%. From the first reference year (2010) to the year with the highest number of published documents (2021), there was an increase of 248.1%, and finally, between 2018 and 2023, there was an increase of 45.28% (Figure 2).

**Figure 3:**
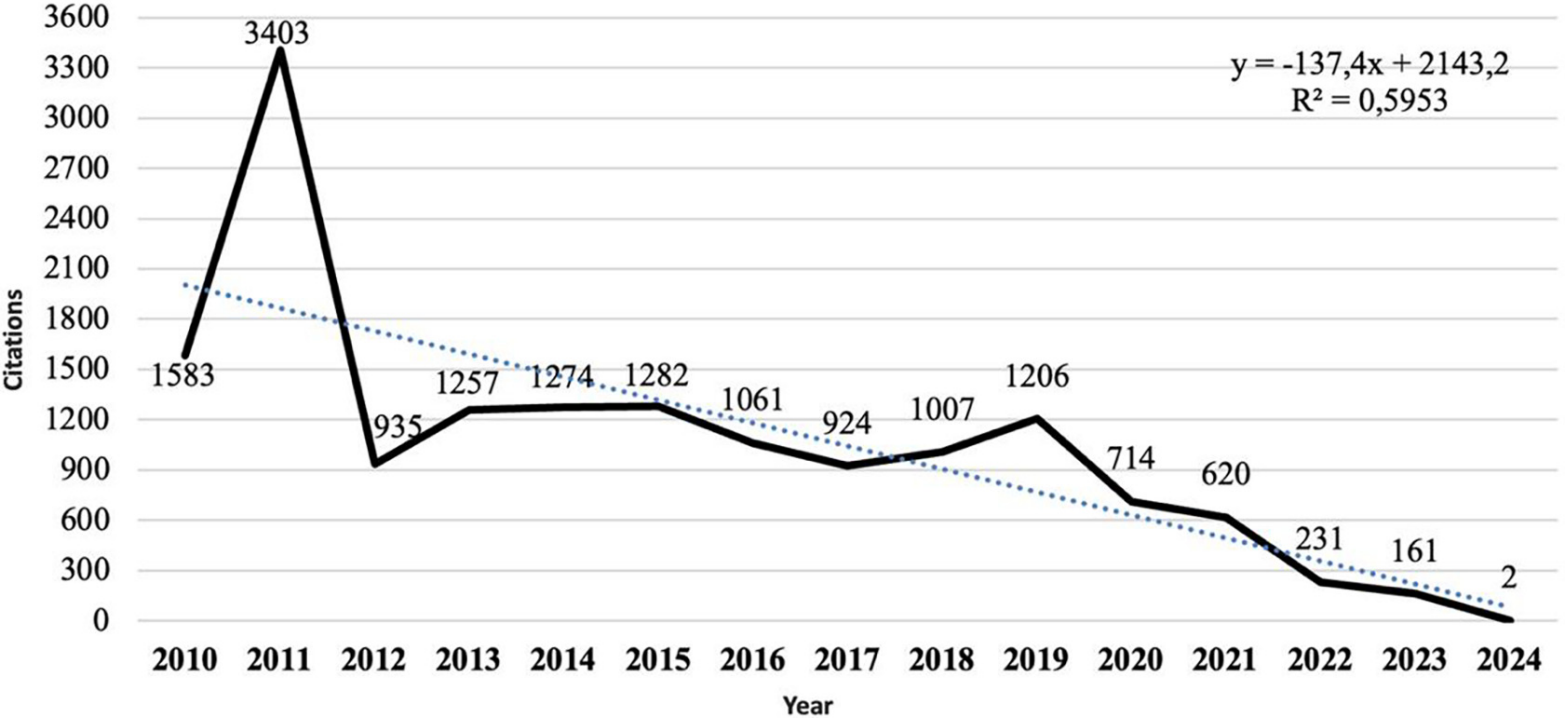
Evolution of the number of annual citations.

In contrast, Figure 3 shows the analysis of citations per year reveals an inverse pattern to that of publications. Between 2010 and 2011, a pronounced increase of 114.97% in citations was observed. Subsequently, a gradual decrease in the annual number of citations occurred, paradoxically coinciding with an increase in the number of published documents. This trend reached its lowest points in 2018 and 2023, with reductions of 70.40% and 86.65% respectively, compared to the years of highest impact (2012 and 2019).

### 3.2 Documents by type

The analysis of methodological designs employed in studies on the 20mSRT test (Table 2) reveals a clear predominance of original scientific articles (95.98%). In contrast, review studies represent a significantly smaller proportion (2.88%), while other document types appear with marginal frequencies. This distribution suggests a research field primarily focused on generating original empirical evidence rather than synthesizing or reviewing existing knowledge.

**Table 2:** Types of published documents.

Document type	Number of documents	Percentage %
Research article	765	95.98
Review article	23	2.88
Conference summary	4	0.50
Letter to the editor	4	0.50
Editorial	1	0.12
Total	797	100

### 3.3 Analysis of the most cited documents

**Figure 4:**
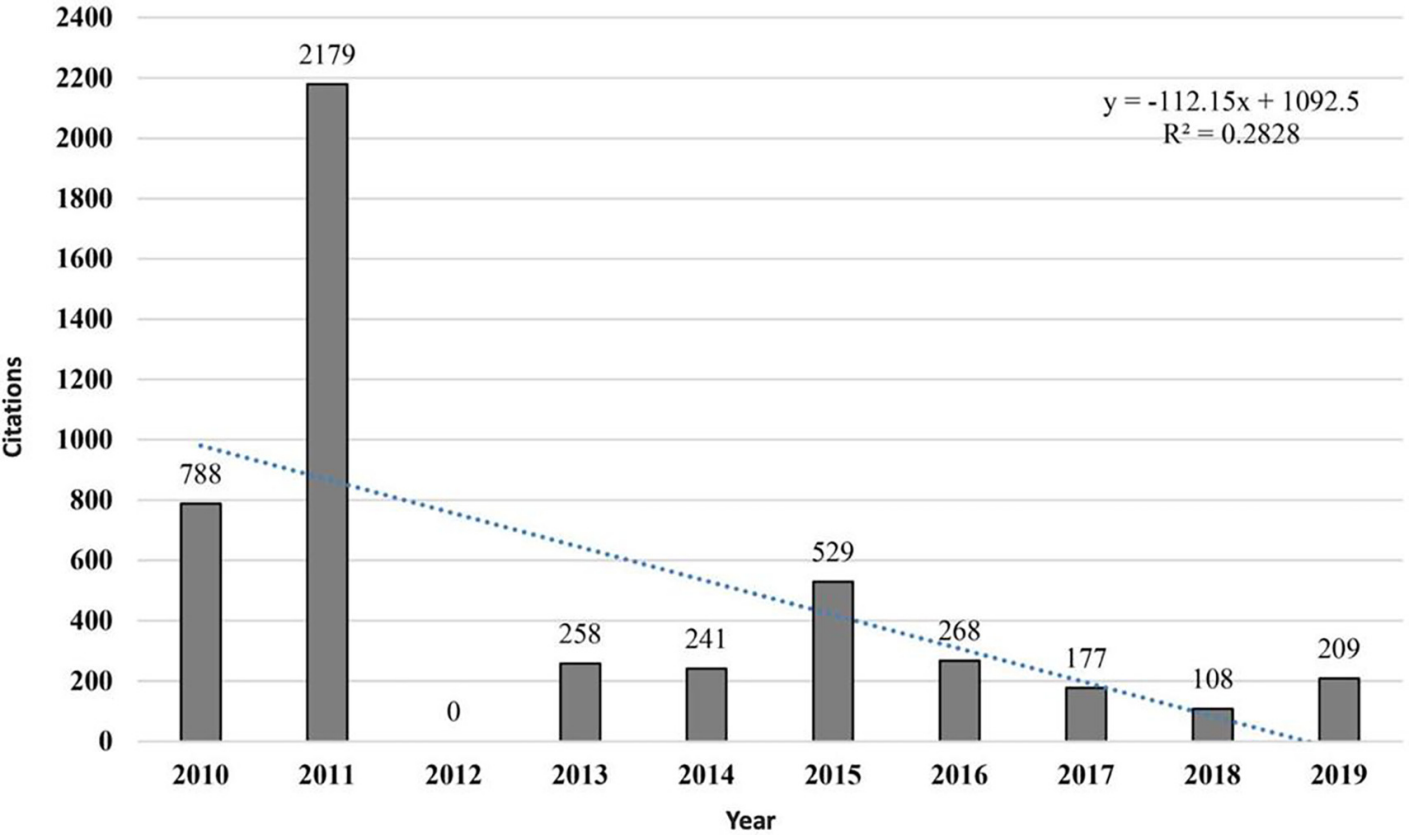
Total number of citations for the 26 studies that have received at least 100 citations.

Only one meta-analysis was identified that studies the 20mSRT (27), as were several systematic reviews addressing the 20mSRT. However, no bibliometric analysis or Scoping Review was found. Figure 4 shows the total number of citations for the 26 studies that have received at least 100 citations. There was a systematic decrease between 2011 and 2015 and between 2015 and 2019.

After the classification and individualized analysis of the documents, Table 3 shows the analysis of review articles that have received at least 100 citations, and Table 4 shows the research articles that have received at least 160 citations. It should be noted that the two most cited studies are review articles: 483 citations (28) and 344 citations (29). Similarly, only one paper, developed by Tomkinson et al. (30), was found at a recent date (last five years).

**Table 3:** Most cited review articles.

Title	Author and publication year	Journal	Objective of the study	Main findings	Total citations	Average citations by year^a^
Field-based fitness assessment in young people: the ALPHA health-related fitness test battery for children and adolescents	Ruiz et al. (2011) (28)	British Journal of Sports Medicine	To summarize the work developed by the ALPHA (Assessing Levels of Physical Activity) study and describe the procedures followed in selecting the tests included in the ALPHA health-related physical fitness test battery for children and adolescents.	When there are time limits, the authors propose the ALPHA health-related fitness test battery, which comprises all evidence-based fitness tests except skinfold thickness measurements. The time necessary to manage this battery to a group of 20 young people by a physical education teacher is less than 2 h. In conclusion, The ALPHA test battery is valid, reliable, feasible, and safe for assessing health-related physical fitness in children and adolescents for population-level health monitoring purposes.	483	37.15
Criterion-related validity of field-based fitness tests in youth: a systematic review	Castro-Piñero et al. (2010) (29)	British Journal of Sports Medicine	To comprehensively study the criterion validity of existing field fitness tests used in children and adolescents.	The results from 73 studies (50 high-quality) addressing the criterion-related validity of field-based physical fitness tests in children and adolescents indicate the following: there is evidence indicating that the 20 m-shuttle run test is a valid test to estimate cardiorespiratory fitness, the hand pressure strength test is a valid measure of musculoskeletal fitness, skinfold thickness, and body mass index are suitable for estimating body composition and waist circumference is a valid measure to estimate central body fat.	344	24.57
Reliability of Field-Based Fitness Tests in Youth	Artero et al. (2011) (31)	International Journal of Sports Medicine	To study the reliability of existing field physical fitness tests intended for children and adolescents.	The review provides an evidence-based proposal for the most reliable field fitness tests for use with children and adolescents, these are: 20 m shuttle run to measure cardiorespiratory fitness; handgrip strength and long jump tests to measure musculoskeletal fitness; 4 × 10 m shuttle run test for height, weight, BMI, skinfold, circumference, and body fat percentage estimated from skinfold thickness to measure body composition.	191	14.69
Criterion-Related Validity of the 20-M Shuttle Run Test for Estimating Cardiorespiratory Fitness: A Meta-Analysis	Mayorga-Vega et al. (2015) (6)	Journal of Sports Science and Medicine	To examine the criterion-related reliability of the 20 m shuttle run tests for estimating cardiorespiratory fitness.	The 20 m shuttle run test has moderate to high validity for estimating cardiorespiratory fitness. The criterion-related validity of the 20 m shuttle run test is significantly higher for adults than for children. However, when the achievement score is combined with other variables, the value of criterion-related validity increases considerably among children. Gender and maximum oxygen consumption level of individuals do not appear to affect the criterion-related validity of the 20 m shuttle test. When maximal oxygen consumption cannot be assessed in laboratory conditions test, the 20 m shuttle run test appears to be a useful alternative to estimate cardiorespiratory fitness.	178	19.77
Systematic Review and Proposal of a Field-Based Physical Fitness-Test Battery in Preschool Children: The PREFIT Battery	Ortega et al. (2015) (32)	Sports Medicine	To systematically review studies conducted in preschool children using field-based physical fitness tests and examine their (1) reliability, (2) validity, and (3) relationship to health outcomes. Our ultimate goal was to propose a battery of field-based physical fitness tests for use in preschool children.	The present systematic review identified the need for further research on the validity of physical fitness tests in preschool-aged children, as well as their relationship with health. Due to this limited information, the PREFIT battery proposed here is based on the results of the present systematic review in preschool children, together with existing evidence in older children and adolescents. While we wait for more evidence to accumulate in preschool children, the PREFIT battery proposed here is a useful tool for assessing physical fitness in children aged 3–5 years.	164	18.22
Physical Exercise Training Interventions for Children and Young Adults During and After Treatment for Childhood Cancer	Braam et al. (2016) (33)	Cochrane Database of Systematic Reviews	To evaluate the effect of a Physical exercise training intervention (at home, in a physical therapy center or in a hospital) on the physical aptitude of children with cancer, in comparation with physical fitness in a control group with usual care.	The effects of exercise training interventions for participants with childhood cancer are still not convincing due to the small number of participants and insufficient study methodology. Despite this, the first results show a trend toward better physical condition in the intervention group compared to the control group. Changes in physical fitness were observed through improved body composition, flexibility, and cardiorespiratory fitness. However, the evidence is limited and these positive effects were not found for the other outcomes evaluated (muscle strength/endurance, daily activity level, health-related quality of life, and fatigue).	161	20.12
Systematic Review of the Relationship Between 20 m Shuttle Run Performance and Health Indicators Among Children and Youth	Lang et al. (2018) (34)	Journal of Science and Medicine in Sport	To summarize the research that assessed the associations between performance on the 20 m shuttle test (20mSRT) and indicators of physiological, psychosocial, and cognitive health among school-age children and youth.	142 studies were identified that determined an association between 20mSRT test performance and a health indicator, representing 319,311 children and youth from 32 countries. The performance of the 20mSRT test was favorably associated with indicators of adiposity and some indicators of cardiometabolic, cognitive and psychosocial health in boys and girls. These findings support the use of 20mSRT as a holistic indicator of population health in children and youth.	108	18.00

BMI, body mass index; m, meters; PREFIT, field-based physical fitness testing in preschool children.

^a^ The average number of citations per year was calculated from the date of publication to July 8, 2024.

**Table 4:** Most cited research articles.

Title	Author and publication year	Journal	Objetive of study	Main findings	Total citations	Average citation by year^a^
Physical Fitness Levels Among European Adolescents: the HELENA study	Ortega et al. (2011) (35)	British Journal of Sports Medicine	To report on sex- and age-specific physical fitness levels in European adolescents.	The figures showed greater physical fitness in boys, except for the flexibility test, and a trend toward greater physical fitness in boys as their age increased, while fitness levels in girls were more stable across of ages.	321	24.69
Objectively Measured Physical Activity and Sedentary Time in European Adolescents the HELENA Study	Ruiz et al. (2011) (36)	American Journal of Epidemiology	To characterize objectively measured levels of physical activity and sedentary time in adolescents from 9 European countries.	A greater proportion of children (56.8% of boys vs. 27.5% of girls) met the MVPA physical activity recommendations of at least 60 min/day. Adolescents spent the majority of recorded time in sedentary behaviors (9 h/day, or 71% of recorded time). Both average intensity and moderate to intense physical activity were higher in adolescents with good cardiorespiratory fitness, and sedentary time was lower in the high physical fitness group. There were no differences in physical activity or sedentary time between maternal education categories.	248	19.07
Temporal Trends in the Cardiorespiratory Fitness of Children and Adolescents Representing 19 High-Income and Upper Middle-Income Countries Between 1981 and 2014	Tomkinson et al. (2019) (30)	British Journal of Sports Medicine	To estimate the international and national temporal trends in cardiorespiratory fitness (CRF) among children and adolescents and examine the relationships between temporal trends in CRF and temporal trends in broad socioeconomic indicators related to health across countries.	Since 1981 there has been a substantial decline in CRF, suggesting a significant deterioration in the health of the population. However, the international CRF trend has not followed the expected trajectory, declining and stabilizing with negligible changes since 2000. CRF data from children in low- and middle-income countries are needed to more confidently determine international trends and determine whether temporal trends are similar to those observed in high- and upper-middle-income countries.	209	41.80
Muscular and Cardiorespiratory Fitness are Independently Associated With Metabolic Risk in Adolescents: the HELENA study	Artero et al. (2011) (37)	Pediatric Diabetes	To examine the independent associations of muscular and cardiorespiratory fitness with pooled metabolic risk in adolescents.	Muscular and cardiorespiratory fitness are independently associated with metabolic risk in adolescents. These results support current physical activity recommendations for youth, which include muscle-strengthening activities in addition to aerobic exercise.	189	14.53
Cognitively Engaging Chronic Physical Activity, However, Not Aerobic Exercise, Affects Executive Functions in Primary School Children: A Group-Randomized Controlled Trial	Schmidt et al. (2015) (38)	Journal of Sport & Exercise Psychology	To investigate the effects of two qualitatively different chronic PA interventions on executive functions in primary school children.	An improvement in shifting performance was found only in the team games and not in the aerobic exercise or control condition. Therefore, the inclusion of cognitive participation in physical activity appears to be the most promising type of chronic intervention for improving executive functions in children, providing further evidence for the importance of qualitative aspects of physical activity.	187	20.77
Alpha-fitness Test Battery: Health-Related Field-Based Fitness Tests Assessment in Children and Adolescents	Ruiz et al. (2011) (39)	Nutrición Hospitalaria^b^	Describe the work developed for the creation of the ALPHA-Fitness field test battery for the evaluation of health-related physical condition in children and adolescents.	The evidence-based ALPHA-Fitness battery includes the following tests: (1) 20-meter shuttle test to assess aerobic capacity, (2) handgrip strength test, and (3) feet-together broad jump test to evaluate musculoskeletal capacity, and (4) BMI, (5) waist circumference, and (6) skin folds (triceps and subscapular) to evaluate body composition. Additionally, 2 variants are included: (i) ALPHA-Fitness high priority battery. This variant includes all the tests except the skinfold measurement, and (ii) the extended ALPHA-Fitness battery, which includes all the tests and in addition to the 4 × 10 m speed and agility test.	181	13.92
Time-motion Characteristics and Physiological Responses of Small-Sided Games in Elite Youth Players: the Influence of Player Number and Rule Changes	Hill-Haas et al. (2011) (40)	Journal of Strength and Conditioning Research	To examine the acute physiological responses and time-motion characteristics associated with 4 soccer-specific small game (SSG) formats (3 vs. 4 players, 3 vs. 3 players + float, 5 vs. 6 players, and 5 vs. 5 players + float) and 4 rule changes in elite youth soccer players.	Subtle changes in SSG game rules can influence physiological, perceptual, and time-motion responses in young elite soccer players. Rules that are related to a team's chances of scoring can improve player motivation and therefore increase training intensity during SSGs. There were no differences between the fixed and variable formats in terms of physiological and perceptual responses, although both can provide useful technical-tactical training. Coaches should be careful when designing different football SSGs, as each rule change or game format can influence exercise intensity independently.	180	13.84
International Normative 20 m Shuttle Run Values from 1 142 026 Children and Youth Representing 50 Countries	Tomkinson et al. (2017) (41)	British Journal of Sports Medicine	To develop sex- and age-specific international standards for the 20 m shuttle run test (20mSRT) in children and youth (ages 9–17) and estimate the prevalence of compliance with the standards referenced in the FITNESSGRAM criteria for healthy cardiorespiratory endurance.	This study provides the most comprehensive and up-to-date set of international sex- and age-specific 20mSRT standards for children and youth, which are useful for health and fitness screening, profiling, monitoring and surveillance.	177	25.28
Effect of Multidimensional Lifestyle Intervention on Fitness and Adiposity in Predominantly Migrant Preschool Children (Ballabeina): Cluster Randomised Controlled Trial	Puder et al. (2011) (42)	BMJ-British Medical Journal	To test the effect of a multidimensional lifestyle intervention on aerobic fitness and adiposity in predominantly migrant preschool children.	A multidimensional intervention increased aerobic capacity and reduced body fat, but not BMI, in predominantly migrant preschool children. The intervention included a physical activity program, lessons on nutrition, media use and sleep, and adaptation of the built environment of the preschool classroom. The intervention also produced beneficial effects on body fat percentage, sum of four skinfolds, waist circumference, and motor agility.	167	12.84
Relationship of Physical Activity With Motor Skills, Aerobic Fitness and Body Fat in Preschool Children: a Cross-Sectional and Longitudinal Study (Ballabeina)	Bürgi et al. (2011) (43)	International Journal of Obesity	To investigate the relationship of objectively measured physical activity (PA) with motor skills (agility and balance), aerobic fitness, and body fat percentage in young children.	Physical activity was positively associated with motor skills and aerobic capacity at baseline, as well as their longitudinal changes. Specifically, only vigorous physical activity, but not total or moderate physical activity, was related to changes in aerobic capacity. In young children, baseline physical activity was associated with improvements in motor skills and aerobic fitness, an important determinant of cardiovascular risk.	160	12.30

^a^ Average citations per year were calculated from publication date to July 8, 2024.

^b^ Document published in Spanish.

A total of 797 documents were identified and included in this review. To conduct the citation analysis, we established a minimum threshold of 50 citations per document.

**Figure 5:**
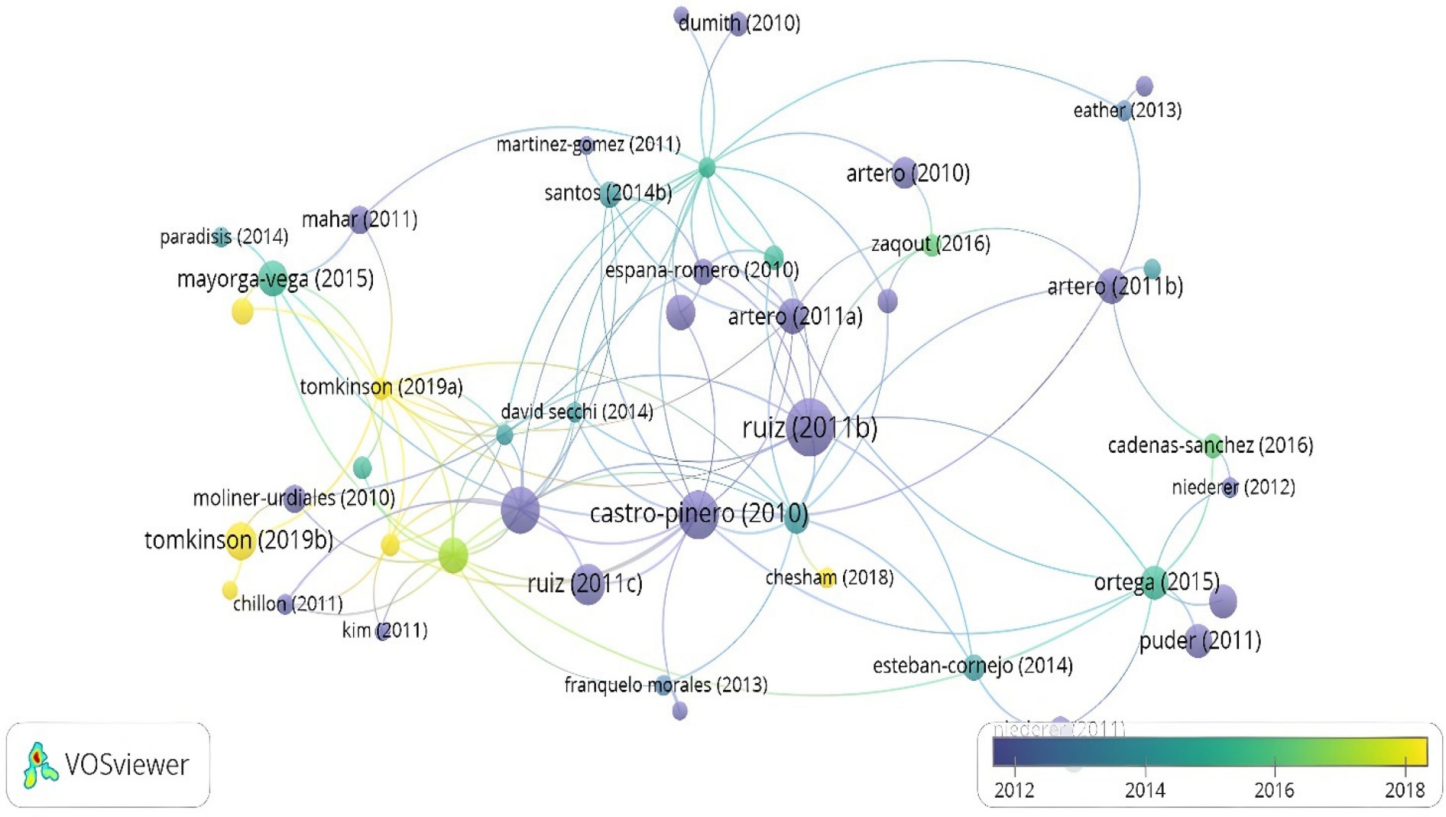
Citation by documents cited at least 50 times.

Among them, only 66 documents (8.28%) exceeded this predefined threshold, and of these, only 47 (71.21%) presented bibliometric interconnections (Figure 5). Chronological analysis of the citation network revealed the formation of 8 main nodes distributed across three distinct temporal periods. During the 2012–2014 period, the highest-impact documents corresponded to Ruiz et al., Ortega et al., Castro-Piñero et al., Ruiz et al., and Artero et al. Throughout the 2014–2016 interval, citations to Mayorga-Vega et al., Ortega et al., and Bianco et al. predominated. In the most recent period analyzed (2016–2018), the documents that accumulated the greatest number of citations were the works of Tomkinson et al. and Lang et al. This temporal distribution reflects the evolution of scientific interest in different dimensions of the 20mSRT.

### 3.4 Authoŕs analysis

For the analysis of the documents by author considering the first author, it is evident that none of the authors has developed more than 10 studies. Thus, the main 16 authors produced 9.91% of the total scientific production. Furthermore, at least two authors have published their studies in a language other than English, and curiously, they are two of the three authors with the highest number of citations for those documents where they appear as first authors (Table 5).

**Table 5:** Number of documents per author taking into account the first author.

Author	Number of publication	Language of publication	Total number of citation received
Ramírez-Vélez, R	8	English	55
Nevill, AM	7	English	38
García-Hermoso, A	7	English	36
Lockie, RG	6	English	35
Cadenas-Sánchez, C	6	English-Spanish	60
Jiménez-Pavón, D	5	English	66
Aandstad, A	4	English	12
Ruiz, JR	4	English-Spanish	61
Gerber, M	4	English	49
Artero, EG	4	English	49
Castro-Piñeros, J	4	English	29
Tomkinson, GR	4	English	23
Machado-Rodríguez, AM	4		
Martínez-Gómez, D	4	English	45
Mora-González, J	4	English	17
Sandercock, GRH	4	English	16
Total	79/797	20.23/100%	

With respect to academic cooperation evaluated in response to the number of authors, it is evident that the largest number of documents, close to half of the total scientific production, is produced between 3 and 5 authors. The production greater than or equal to nine authors is greater than that produced by authors 1–2, highlighting the importance of collaboration between researchers to produce new knowledge in the 20mSRT study (Table 6).

**Table 6:** Total number of documents by author.

Number of authors	Number of studies	Percentage %
1–2	48	6.02
3–5	327	41.02
6–8	278	34.88
≥9	144	18.06

**Figure 6:**
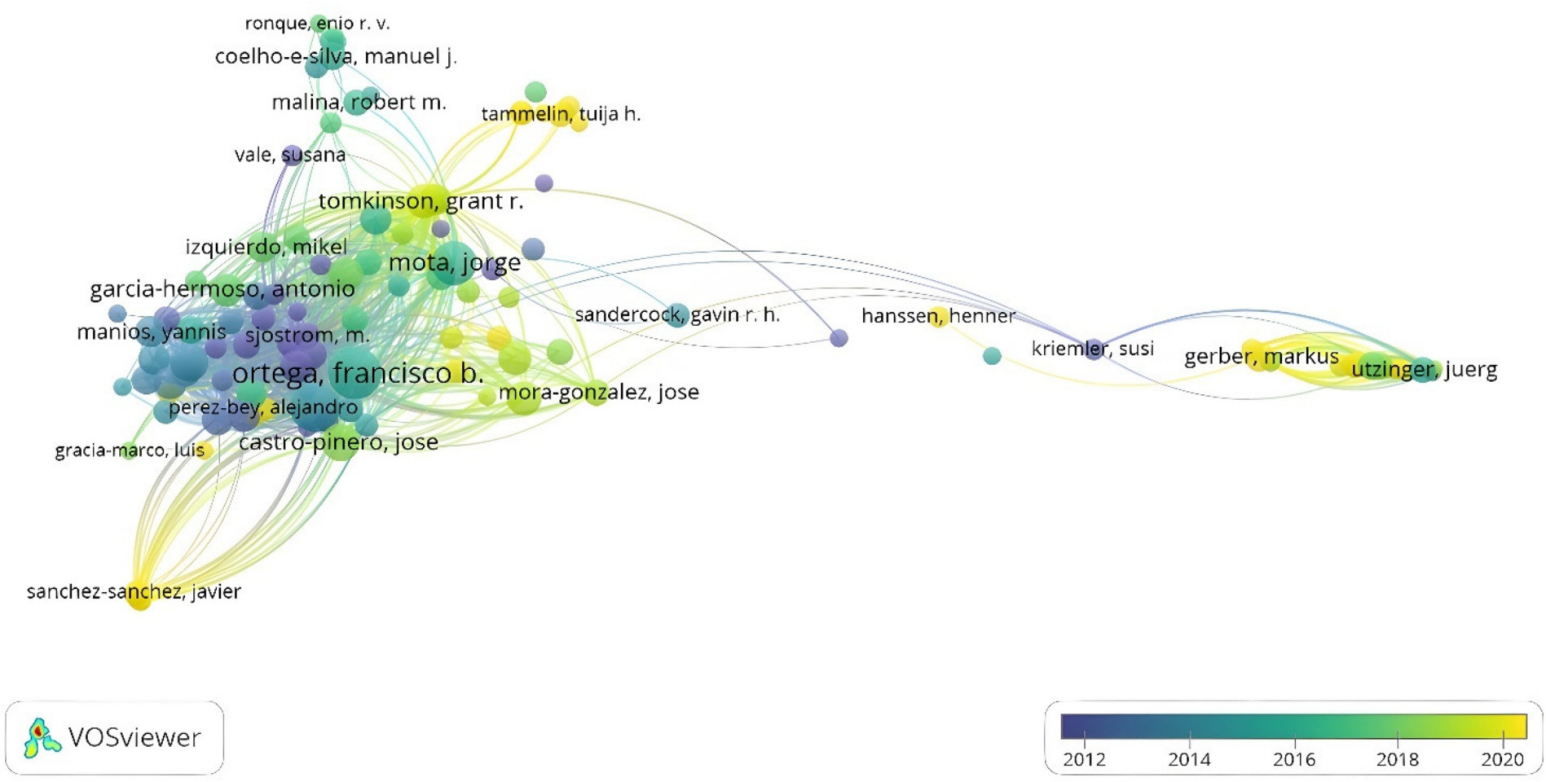
Citation by authors.

Figure 6 shows the interactions produced between the citations of the authors. The size of the nodes represents the number of published documents, and the color corresponds to the established connections. A strong concentration is observed from 2012 to 2014 led by Ortega, F., whereas Tomkinson, G., has led the academic contribution between 2018 and 2020. Similarly, there are other nodes that are not closely related, which respond to a smaller number of citations produced: Kriemer, S., Utzinger, J., and Gerber, M.

### 3.5 Keyword analysis

**Figure 7:**
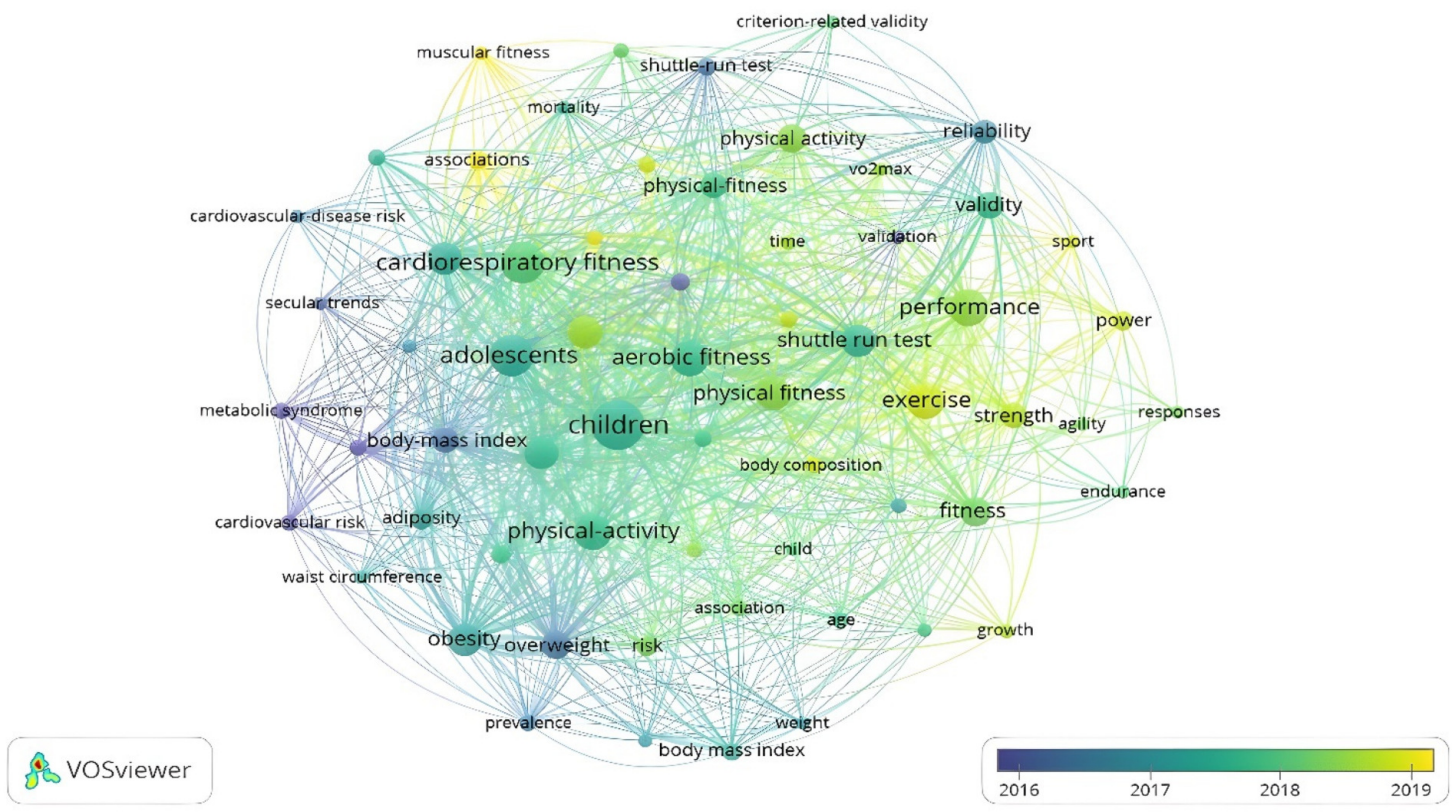
Occurrences by keywords in response to temporality.

For occurrence by keywords, it was established that the word was included at least 20 times in each study. Thus, of the 2,546 words identified, only 63 met the threshold. The analysis of the most recurrent keywords in the studies that analyze the 20mSRT reveals that these keywords are diverse in reference to the age of the participants and are associated with other physiological and physical condition variables. Depending on time, for 2016, the most referenced concepts were “cardiovascular risk”, “metabolic syndrome”, and “overweight”. From 2017 to 2018, the concepts used were “children”, “adolescents”, “cardiorespiratory fitness”, “aerobic fitness”, and “valence”. In 2019, the most referenced concepts were “exercise”, “muscular fitness”, “strength”, “body composition” and “association”. Thus, the most recent concepts are associated with nutritional and physiotherapy processes, among which the following stand out: “supplementation”, “ingestion”, “prevention”, “risk” and “concurrent training” (Figure 7).

**Figure 8:**
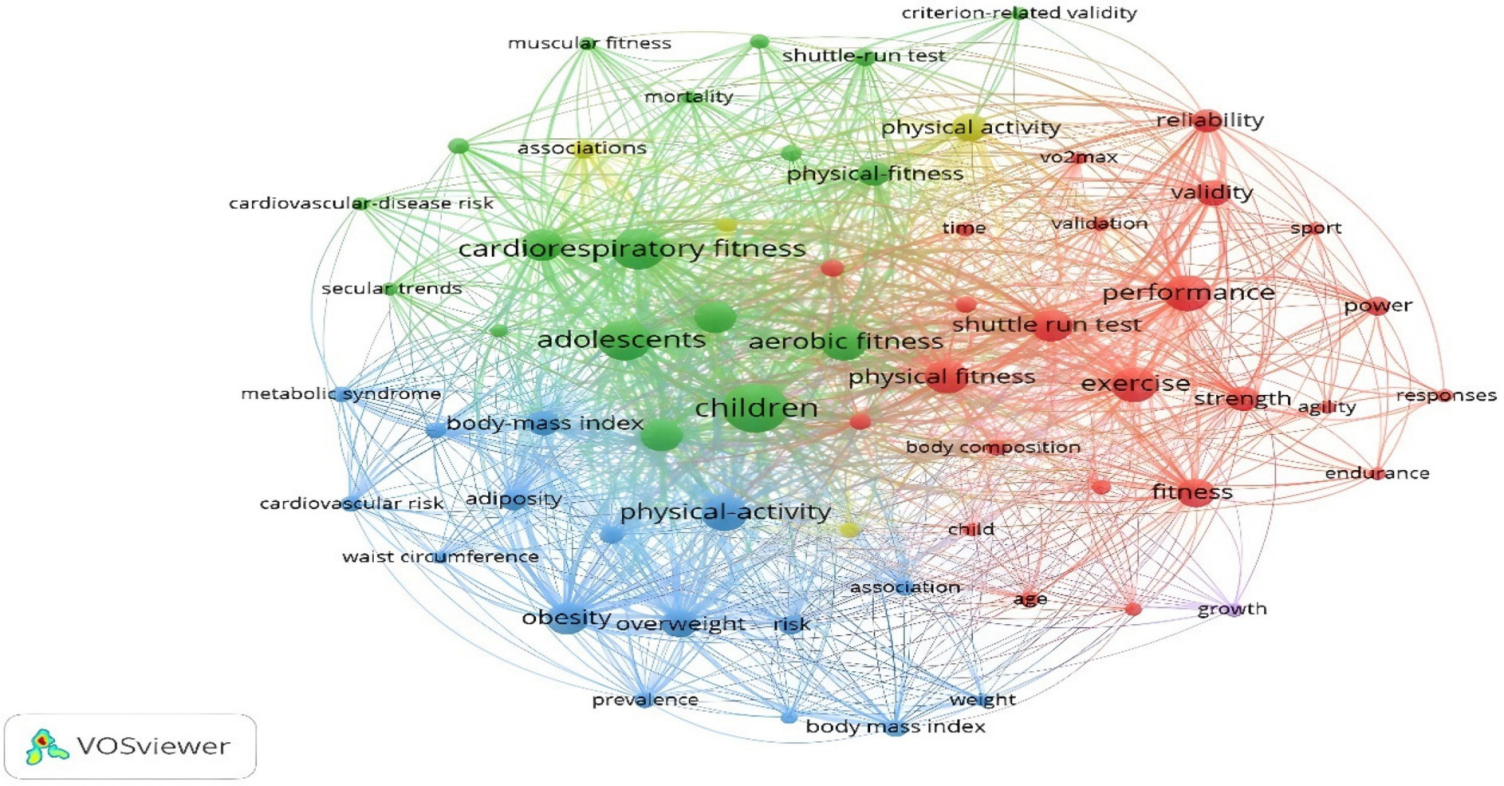
Occurrences by keywords.

Moreover, the concepts related to the number of times that have been cited the most are children (*n* = 290), cardiorespiratory fitness (*n* = 205), adolescents (*n* = 203), performance (*n* = 164), aerobic fitness (*n* = 146), exercise (*n* = 145), physical activity (*n* = 142), physical fitness (*n* = 134), obesity (*n* = 128), childhood (*n* = 126), youth (*n* = 125) and health (*n* = 125) (Figure 8).

### 3.6 Analysis of the journals and cited references

**Figure 9:**
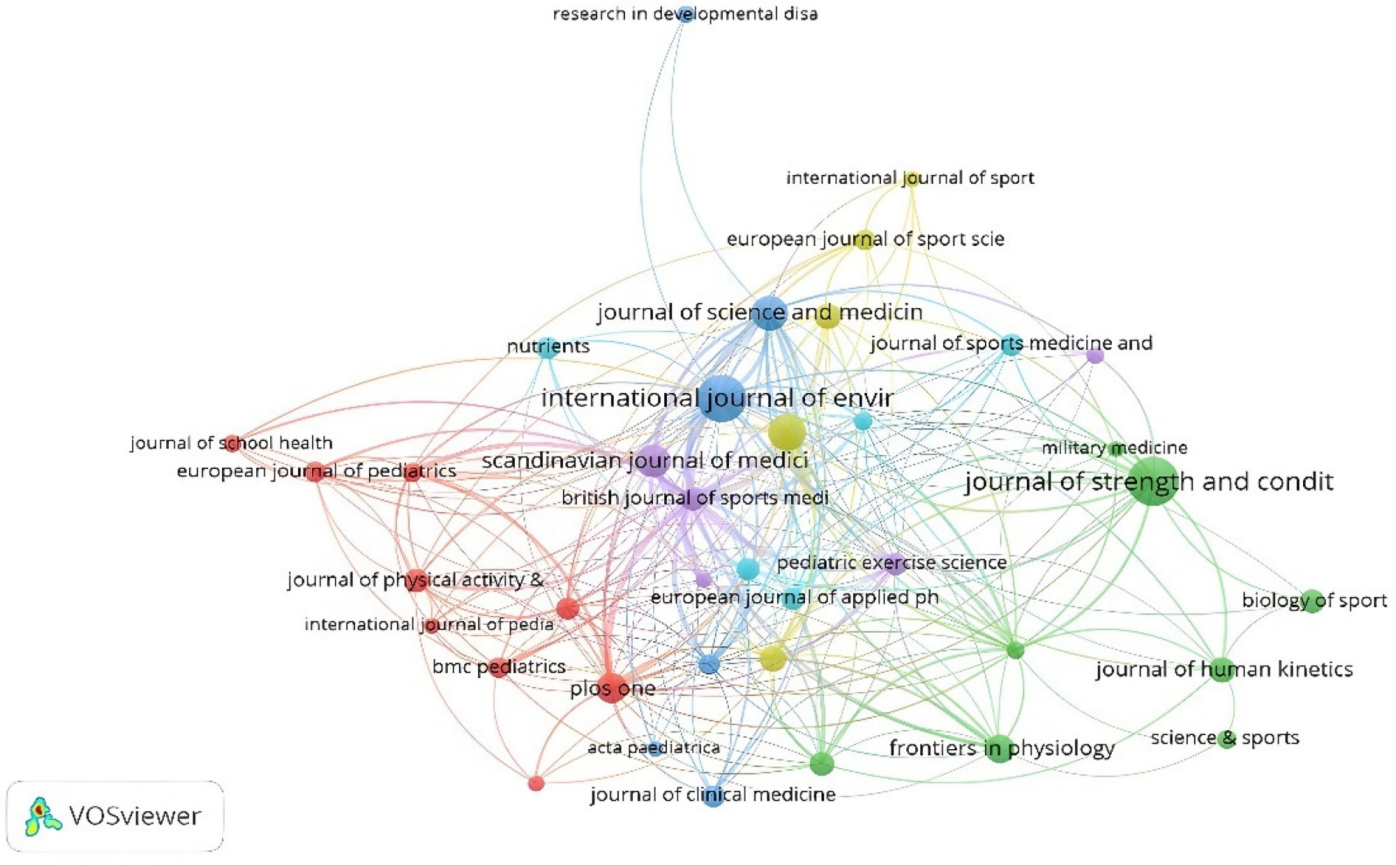
Citation by journals.

There are 243 journals that published the 797 documents found for this bibliometric review. To analyze citations by journal, the journal was considered to have published at least five documents and had received at least 20 citations. Thus, only 38 journals met the threshold. Similarly, the citation map by journal shows that the journals with the greatest number of published documents are the Journal of Strength and Conditioning Research (*n* = 45), the International Journal of Environmental Research and Public Health (*n* = 42) and the Journal of Sports Sciences (*n* = 25). The colors represent the established connections. Each node is defined by a color. In this case there are at least 6 nodes, with the red node led by Plos One and the green node led by Journal of Strength and Conditioning Research being the most important (Figure 9).

**Figure 10:**
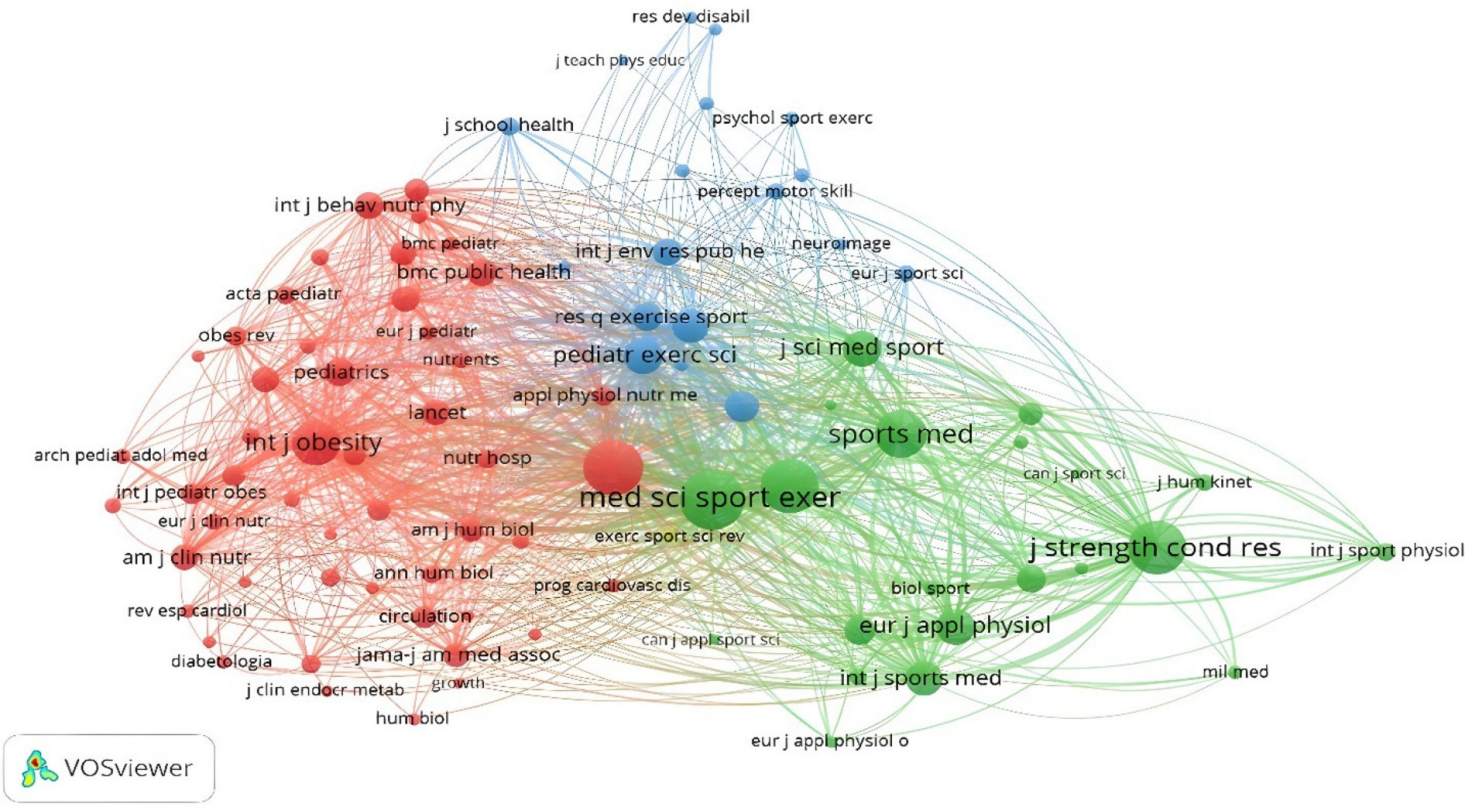
Cocitation by journals.

Similarly, for the analysis of cocitations between the journals, 5,565 sources were found, where it was established that the minimum number of citations for each journal was 50. Only 90 journals met the requested threshold. The journals with the highest number of citations are Medicine and Science in Sports and Exercise (*n* = 1,359) and the British Journal of Sports Medicine (*n* = 1,143). The colors represent the established connections. Each node is defined by a color. Three main nodes are established, with the green node led by Sport Medicine and the red node led by Medicine & Science in Sports & Exercise (Figure 10).

**Figure 11:**
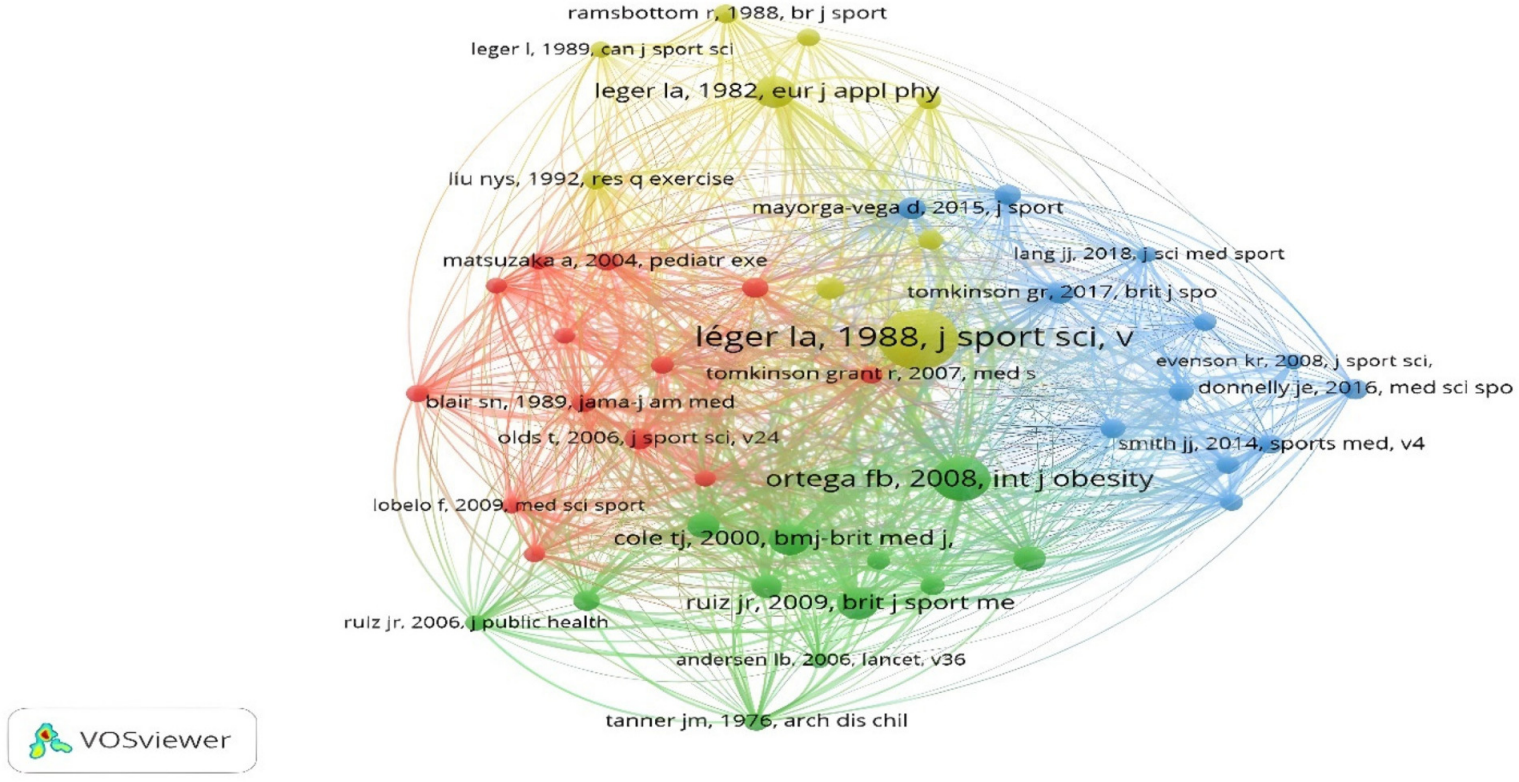
References cited.

For the analysis of the cited references, 20.074 were included, of which 46 were cited at least 30 times. The studies of Leger, Cole, Ortega and Ruiz stand out. The colors represent the established connections. Each node is defined by a color. Four major nodes are established, with the yellow and green nodes being the most relevant. The yellow node is led by the study of Leger and the green node by Ortega (Figure 11).

### 3.7 Analysis of editorial groups

The scientific production carried out by the publishers that disseminate scientific knowledge about the 20mSRT study reveals how the Multidisciplinary Digital Publishing Institute (MDPI) is the editorial group with the largest number of published documents, although it is not the one that receives the largest number of citations. The first six editorials represent half of the total scientific production (49.43%). There are 4 publishers that have received more than 1,000 citations (Table 7).

**Table 7:** Total number of documents published by editorials.

Name	Number of documents	Number of citations
MDPI	100	701
Wiley	73	1,406
Elsevier	65	1,367
Lippincott Williams & Wilkins	64	1,433
Taylor & Francis	47	711
Springer	45	875
BMC	33	632
Human Kinetics Publ INC	30	742
BMJ Publishing Group	25	2,564
Frontiers Media SA	25	159
Edizioni Minerva Medica	22	128
Oxford University Press	12	504
Sage Publications Ltda.	12	147
Total: 13 editorials	553/797	11,369

MDPI, Multidisciplinary Digital Publishing Institute; Wiley, Wiley Online Library; BMC, BioMed Central.

### 3.8 Areas of knowledge

The analysis of the areas of knowledge where documents on 20mSRT are published reveals that the most prolific and cited areas are sport sciences, with an average of 25.19; public, environmental & occupational health, with 22.96; and pediatrics, with 17.20 citations per published document. Similarly, the main one is “Sport Sciences”. Similarly, the first five areas covered more than half of the published documents (56.58%) (Table 8).

**Table 8:** Areas of knowledge.

Areas^a^	Number of publications	Number of citations
Sport Sciences	263	6,627
Pediatrics	88	1,514
Public, Environmental & Occupational Health	56	1,286
Medicine, General & Internal	54	870
Environmental Sciences	44	319
Nutrition & Dietetics	42	835
Physiology	34	590
Multidisciplinary Sciences	34	475
Hospitality, Leisure, Sport & Tourism	20	364
Endocrinology & Metabolism	19	842
Psychology	19	177
Education and Educational Research	16	129
Anthropology	15	234
Health Care Sciences & Services	10	153
7 areas	714/797	14,415

^a^ The same article can be considered in more than one area.

### 3.9 Language and analysis by country

**Figure 12:**
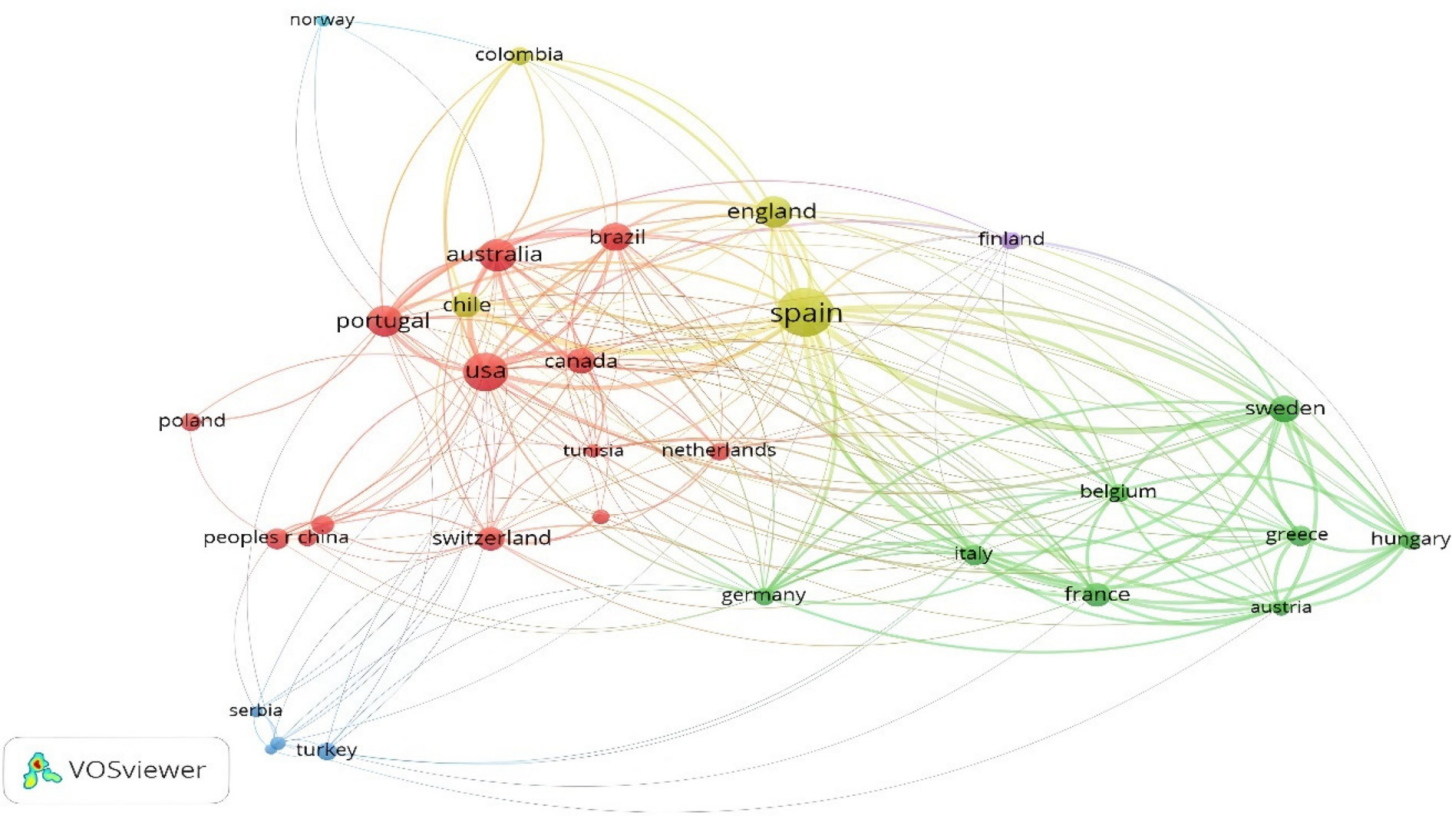
Coauthorship by country.

The analysis by language shows that the greatest production is generated in the English language (*n* = 773), with 96.98% of the total scientific production, followed by Spanish (*n* = 17), with 2.13%. Furthermore, among the 797 documents reviewed and included in the present bibliometric study, three documents were found in Portuguese, two in German, one in French and one in Russia. Figure 12 shows coauthorship by country. It details how there are three main cooperation networks: the yellow network led by Spain and with the largest number of connections with other countries; the red network led by the connections between the United States, Australia, Portugal, Canada and Brazil; and, finally, the green network led by Sweden and France. The colors represent the established connections. Each node is defined by a color. For the countries, five nodes are established, with yellow and red being the most relevant. The yellow node is led by Spain and England, while the red node has the largest number of countries, led by USA, Canada, Portugal, Australia and Brazil.

**Figure 13:**
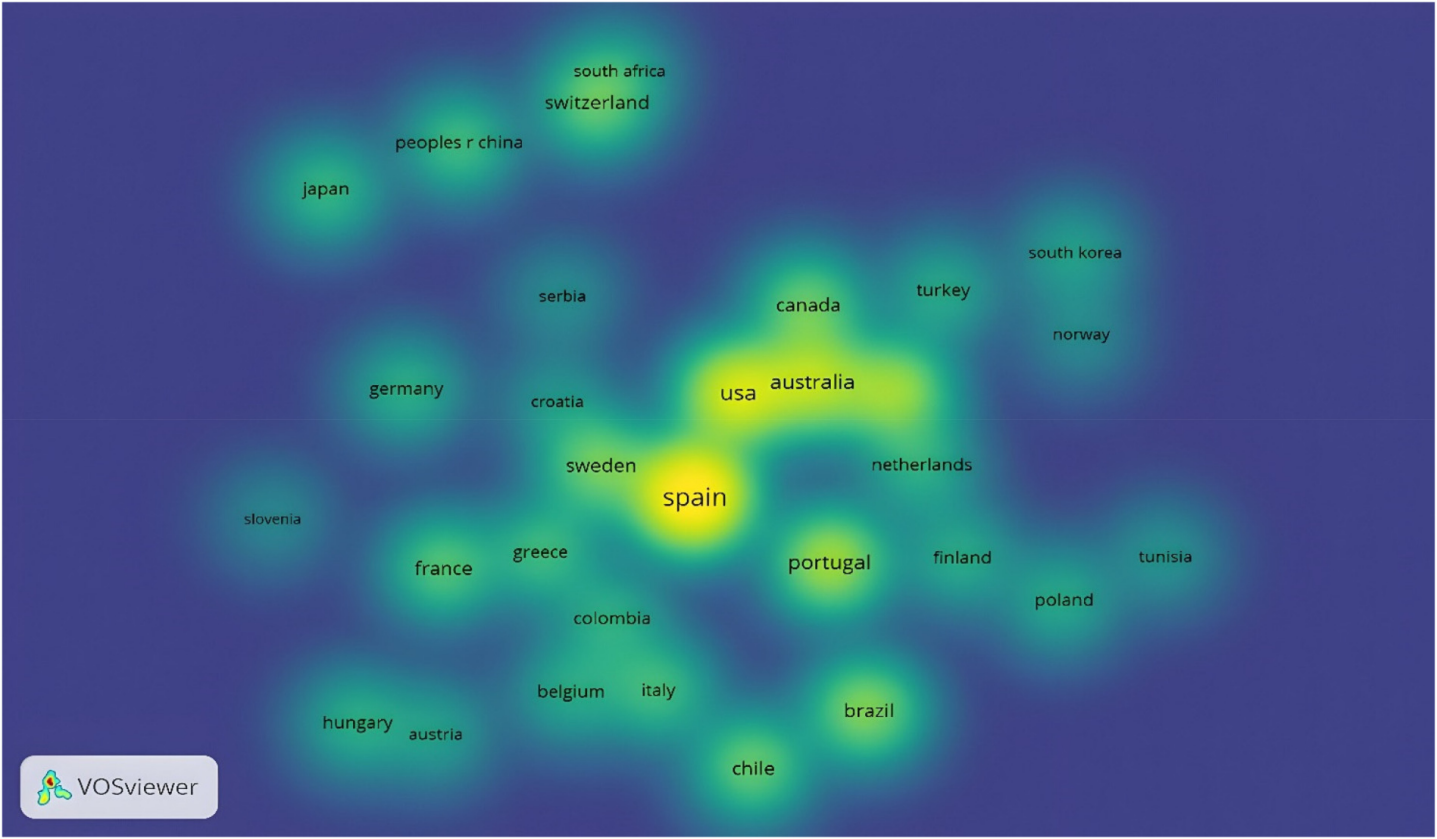
Citation by countries.

Figure 13 shows the coauthorship and citations of documents by country. There, it is evident that only 84 countries have contributed to the knowledge of 20mSRT. To establish the threshold, it was determined that each country had published at least 10 studies and received 50 citations. Thus, only 31 countries met the threshold. The countries that receive the greatest number of documents and citations are Spain (*n* = 187 documents and 6,209 citations), the United States (*n* = 112 documents and 3,475 citations), Australia (*n* = 85 documents and 2,393 citations) and Sweden (*n* = 54 documents and 4121 citations). The map shows that Spain is the epicenter country of all the connections that are established with the subject, whereas there are countries that are increasing their scientific production in European countries (Serbia, Poland, Slovenia and Croatia), Asian countries (China, Japan and South Korea), Africa (Tunisia) and South America (Chile). Finally, there are countries from each continent, which highlights the importance of this topic among the academic community.

### 3.10 Organizations

For organizations, there are 1,174 organizations that have been studying the 20mSRT. Only 38 organizations published at least 10 studies and received at least 50 citations. Specifically, the University of Granada is the organization that has the most publications and citations (*n* = 83 and 4,631), the Karolinska Institute (*n* = 46 and 3,880), the University of Zaragoza (*n* = 37 and 2,346) and the University of Cadiz (*n* = 34 documents and 2,106) of which 59 comply with 2 documents and two subpoenas. Of the 59 organizations, only 33 appear to be connected.

**Figure 14:**
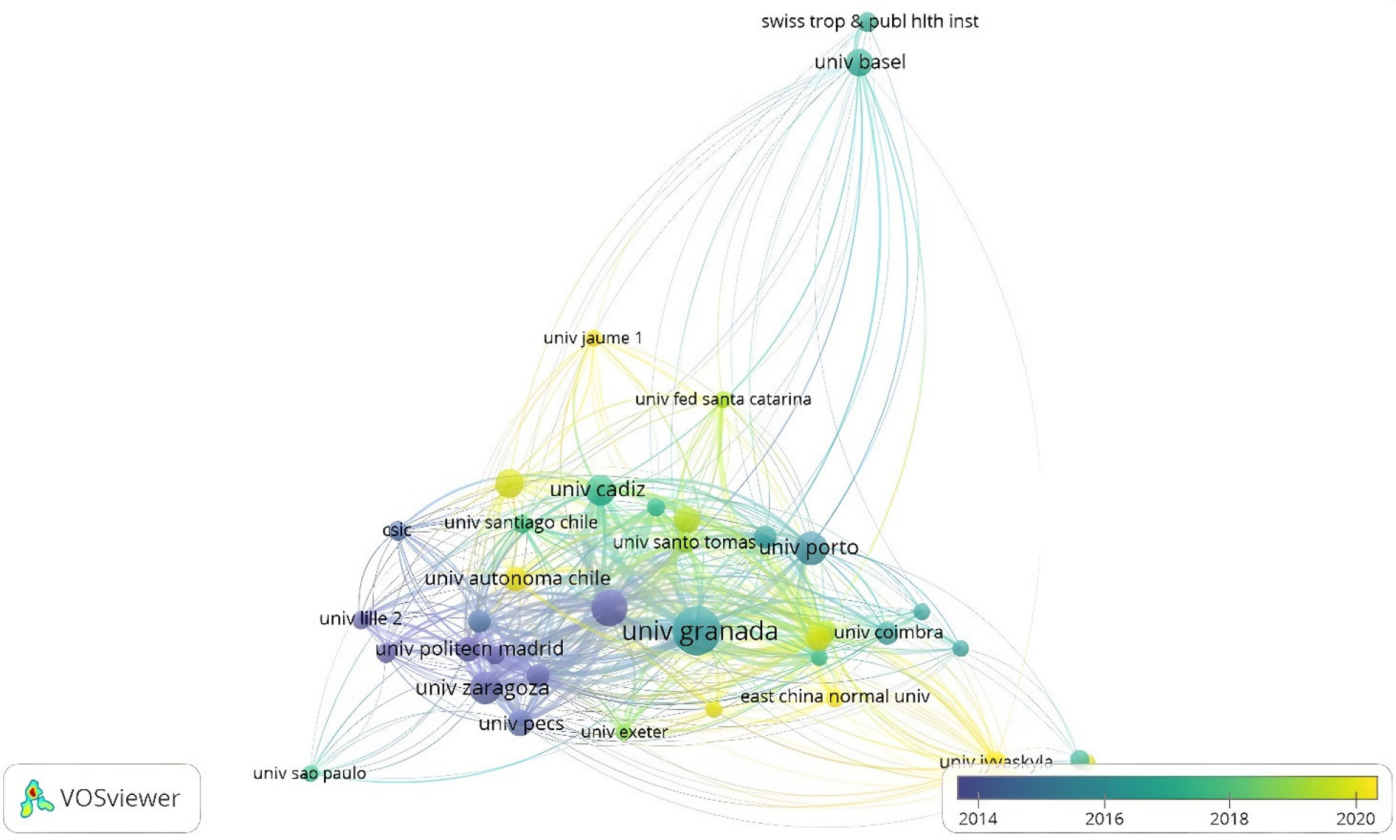
Coauthorship by organizations.

With respect to time (2014–2016), a strong trend is evident in Spanish universities, among which the University of Zaragoza and the Polytechnic University of Madrid stand out. For the years 2016–2018, there was an increase in the number of documents and networks from different universities, where the University of Granada and Cadiz in Spain, Basel University in Switzerland, University of Coimbra and Porto in Portugal and, finally, University of Santiago stand out from Chile. For the years 2018–2020, there are organizations from other continents that are carrying out various studies, including the Autonomous University of Chile and the Federal University of Santa Catarina in South America, East China Normal University in Asia, University Jyväskylä and Universitat Jaume I in Europe, on the production of knowledge related to the 20mSRT (Figure 14).

### 3.11 Evaluated samples

There was diversity in the samples evaluated when the 20mSRT was used, highlighting its application in children and adolescents, with more than half of the total scientific production (58.71%). It also highlights its applicability in the evaluation of athletes (Table 9).

**Table 9:** Characteristics of the evaluated sample.

Areas*	Number of publications	Percentage (%)
Children	249	31.24
Adolescents	219	27.47
Athletes	142	17.81
Children and adolescents	75	9.41
Adults	64	8.03
Military forces	21	2.63
Adolescents and adults	10	1.25
N/A	6	0.75
Children and adults	3	0.37
Firemen	3	0.37
Athletes and no athletes	2	0.25
Children, adolescents y adults	2	0.25
Referees	1	0.12

^a^ The same article can be considered in more than one Sport.

The characteristics of the athlete population also highlight concerns for different sports when the 20mSRT is used to assess cardiorespiratory fitness. It has been used in 23 sports, in athletes with disabilities and in team and individual sports (Table 10).

**Table 10:** Characteristics of the athlete sample evaluated with the 20mSRT.

Sport*	Number of publicacions	Percentage (%)
Soccer	40	28.16
Handball	13	9.15
Basketball	11	7.74
Australian futbol	9	6.33
Tennis	8	5.63
Athletics	7	4.92
Judo	7	4.92
Badminton	5	3.52
Rugby	5	3.52
Taekwondo	5	3.52
Karate	4	2.81
Rowing	4	2.81
Hockey	4	2.81
Volleyball	4	2.81
Team sport	3	2.11
N/A	3	2.11
Futsal	2	1.40
Basketball on wheelchair	1	0.70
Ciclying	1	0.70
Gimnasy	1	0.70
Wrestling	1	0.70
Swimming	1	0.70
Netball	1	0.70
Pentathlon/	1	0.70
Alpine ski	1	0.70
Total	142	99.99/100

^a^ The same article can be considered in more than one Sport.

## 4 Discussion

To our knowledge, this is the first study that performs a bibliometric analysis of scientific publications on the use of 20mSRT to evaluate cardiorespiratory fitness (CRF) in sports sciences oriented to sports performance and health. The number of annual publications on this topic shows exponential growth, in line with other bibliometric studies that analyze the global panorama of physical literacy research (44), medical research (45) and research based on child health surveys (46). To this end, a bibliometric analysis of the studies analyzed in the WoS and PubMed databases was carried out. The main findings were as follows:

(1) A total of 797 documents related to the 20mSRT study have been published.
(2) The number of studies increased by 185.18% between 2010 and 2023, and, in turn, the years with the greatest production were 2020, 2021 and 2022.
(3) There was a preference for the development of research articles (95.98%), with a very low percentage of review studies.
(4) As of 2010, only one meta-analytic study has been reported, and no scoping review or bibliometric analysis has been found to date in the document search.
(5) at least 26 studies that have received 100 citations.
(6) There is a decrease in the number of citations received from studies that have 20mSRT as their study topic, mainly between 2011 and 2015 and 2015–2019.
(7) 2011 was the year with the highest number of citations, and starting in 2020, there was a decrease in total annual citations.
(8) The main 16 authors produced 9.91% of the total scientific production.
(9) the largest number of documents, close to half of the total scientific production, is produced between 3 and 5 authors.
(10) The concepts with the greatest occurrence are Children, Cardiorespiratory Fitness, Adolescents, Performance, Aerobic Fitness, Exercise, Physical-Activity, Physical Fitness, Obesity, Chilhood, Youth and Health.
(11) 243 journals have published the 797 papers, and the top three journals that have published manuscripts related to 20mSRT are: Journal of Strength and Conditioning Research (USA), International Journal of Environmental Research and Public Health (Switzerland) and Journal of Sports Sciences (UK).
(12) The MDPI is the publishing group with the largest number of published documents, and those that receive the largest number of citations are BMJ Publishing Group and Lippincott Williams & Wilkins.
(13) Sport Sciences, Public, Environmental & Occupational Health and Pediatrics are the main areas in which studies are published according to WoS categories.
(14) the prevailing language in the publications is English.
(15) the most prolific countries in terms of the number of documents and subpoenas received are Spain, the United States, Australia, Sweden and Portugal.
(16) According to the density map, there are countries for each continent, which demonstrates the importance of this topic among the international academic community.
(17) the main organizations that share their research are Spanish, with the University of Granada, University of Zaragoza and University of Cádiz being the ones that develop the greatest number of studies.
(18) There is diversity in the samples evaluated, ranging from studies in children, adolescents and adults to studies evaluating physical conditions, military forces, firefighters, and referees to athletes seeking to determine the effects on specific abilities.
(19) In response to the sport in which the 20mSRT test is used, those with the greatest number of studies are team sports: soccer, handball, and basketball.

The results reported by this analysis can be used by researchers to evaluate the scientific interest of using 20mSRT in different population groups. Other studies have referred to the importance of the findings derived from bibliometric analyses, highlighting the value of revealing research trends in a specific field of knowledge and thereby enabling the study of less explored problems (7, 13). The use of the 20mSRT test has been confirmed to occur mainly in the evaluation of cardiorespiratory fitness in children (47, 48), adolescents (49, 50) and adults (51). In turn, few studies have evaluated children and adults in the same investigation. This may respond to the specificities of knowing in deep childhood and adulthood separately. The study developed by Tello-Navarro et al. (52) revealed that by delving deeper into a topic, the research landscape can be expanded, revealing that there are emerging concepts associated with adulthood, adolescence, and young adulthood. Moreover, other studies in adults have revealed that the results of bibliometric analyses could be directed at predicting future research trends, thus helping to promote the understanding of a topic for all the actors included, including those responsible for policies. administrators of economic resources and researchers (53).

An analysis of the 17 most cited studies identified in the present study and that have received at least 100 citations revealed that there is a concern about the evaluation of physical fitness (cardiorespiratory) related to the health of children and adolescents of school age (29, 36, 37) while evaluating the effects of different interventions on motor skills (43) and learning styles. Life and adiposity (42) and in children with cancer (34). This preference toward the study of physical condition in children has also been addressed in other bibliometric review studies, highlighting that studies on physical activity in preschool children have increased significantly between 2024 and 2020, and, in turn, the most studied thematic areas are teaching and health (54). Similarly, the study by Jiménez-Jiménez et al. (54) concluded that the methodological techniques most commonly used to evaluate children's health are questionnaires and accelerometry and that there is a greater preference for the development of experimental studies that use intervention programs with the inclusion of a control group. These findings are related to the results of the present bibliometric study, where there is a greater number of studies and a greater number of citations for those research articles that use the 20mSRT.

On the other hand, one study aimed at evaluating whether improvements in cardiorespiratory fitness, muscle strength and body composition are similar at the onset of obesity in adulthood vs. the onset of obesity in childhood (55). This demonstrates the interest in recognizing physical activity as a facilitator that helps in the primary and secondary prevention of different chronic diseases, including cardiovascular diseases, diabetes, cancer, hypertension, obesity, depression and osteoporosis (56, 57). This is related to the analysis of the most cited documents on the use of 20mSRT in the present bibliometric study because the concern of the most cited research focuses on evaluating children and adolescents and, with it, being able to implement interventions (58), plans and programs that help resolve future sedentary behaviors and inappropriate lifestyles (59).

Its use has also been identified in the evaluation of athletes in practices where there is only 1 study, including Alpine Skiing, Pentathlon, Netball, Swimming, Wrestling, Gymnastics, cycling and wheelchair basketball. The largest number of studies on soccer players has been conducted on the evaluation of adolescents (60), semiprofessionals (61) and professionals (62). With this, the opportunity to be able to evaluate physical conditions in other sports and in response to different levels is also evident: training, specialization, semiprofessional and professional. This is perhaps one of the main contributions of this bibliometric review study that helps to favor the current panorama of research trends and thereby reveals that there are areas, sports, population groups, countries and concepts that need greater research development.

### 4.1 Future perspectives

Given the future perspectives of studies focused on evaluating, identifying and recognizing the contributions of 20mSRT to evaluate physical conditions, specifically cardiorespiratory fitness in different population samples, the need arises to carry out a greater number of review studies, including meta-analyses, scoping reviews and more specific bibliometric analyses. Similarly, in the evaluation of athletes, a greater number of randomized controlled trials, longitudinal designs and case studies need to be developed that provide a greater understanding of the effects on other physical, technical, tactical abilities, etc. Faced with this, it is necessary that scientific evidence allows us to glimpse the effects that different training protocols have on the adaptations induced for other sports that were not identified in the present study.

The use of the 20mSRT has been increasing in the number of studies that use it for physical assessment in heterogeneous population samples; however, very few studies with multivariate designs exist that allow us to understand the relationships established between cardiorespiratory fitness and other sociodemographic characteristics. psychosocial, educational, emotional, etc., of the evaluated individuals.

### 4.2 Limitations

The present study presents different limitations associated with the heterogeneity of population samples and the characteristics evaluated, as well as the name of the test. Similarly, the present study was limited to documents cataloged in the Web of Science and PubMed, so the language biases of these indexers must be considered. An additional limitation of this study is that the search was carried out on the basis of the following concepts: “20-m shuttle run test”, “20-m shuttle run test”, “20 m shuttle run”, “20 m SRT”, “20-m endurance shuttle run test”, and “20-m multistage shuttle run test”, excluding terminology related to “Course-navette” and “Navette test”.

## 5 Conclusions

The specialized literature in the study of the 20mSRT has a preference for the study of physical conditions in different populations, highlighting its use in children and adolescents, with a smaller proportion of adults, athletes and military forces. Studying the most cited studies confirms the need for more review research. The countries that have led scientific production are the European continent (Spain and Sweden), North America (the United States) and the Oceanic continent (Australia); however, the institutions that have been studying the subject the most are Spanish. Moreover, various countries have increasingly published studies related to 20mSRT, highlighting those from the European, Asian, African and South American continents, revealing the interest in this topic among the academic community.

## Data Availability

Publicly available datasets were analyzed in this study. This data can be found here: The data can be requested from the corresponding author.
